# GOALS OF THE ENDGAME

**DOI:** 10.1093/geroni/igz038.786

**Published:** 2019-11-08

**Authors:** Michelle Silver

**Affiliations:** University of Toronto, Toronto, Ontario, Canada

## Abstract

At all stages of life, the body can be considered an occupational resource that interacts with social structures in identity formation and complicates personal adaptation to life transitions. As the body declines, the economic and social standing it confers also tends to decline, leading to socially embedded fears about physical decline and marginalization. This paper applies theoretical work from embodiment theory and the life course perspective to examine how perceptions of aging and life experience with sport (or lack thereof) influence exercise participation and athletic identity. Using a narrative approach, I examine in-depth interviews I conducted with elite athletes, masters’ athletes, coaches, athletic program directors, mature adults. Some participants struggled to exercise regularly, and others are exercising more in their later years than at any other point in their lives. Three key themes emerged: 1) bodily identity is tremendously important in relation to other forms of identity when it is affected by aging, ill-health, or other physical processes; 2) physical functional mobility becomes increasingly important with age; and 3) experiences with sports and athletic identity (or lack thereof) influence engagement in exercise in later life in surprising ways. The paper challenges society’s focus on youth in sports and elite athletes, to discuss how our greater longevity means that we must place more emphasis on identifying ways to keep physically active and mobile throughout adulthood.

